# Irritable bowel syndrome and its impact on utilization and outcomes in elective thoracolumbar fusion^☆^

**DOI:** 10.1016/j.bas.2026.106087

**Published:** 2026-05-05

**Authors:** Chiemela Izima, Farhan A. Khan, Bhargav Ayloo, Joshua S. Fuller, Nathan A. Shlobin, Isaac S. Lee, Dean Chou, Andrew K. Chan

**Affiliations:** Department of Neurological Surgery, Columbia University, New York, NY, USA

**Keywords:** Functional gastrointestinal disorders, Ileus, Irritable bowel syndrome, Perioperative outcomes, Spinal fusion, Thoracolumbar spine

## Abstract

**Introduction:**

Functional somatic syndromes have been associated with worse outcomes, but the impact of irritable bowel syndrome (IBS) remains unclear.

**Research question:**

Is IBS associated with perioperative complications following thoracolumbar spinal fusion?

**Materials and methods:**

The 2021-2022 National Inpatient Sample was reviewed for adults with and without IBS undergoing elective thoracolumbar fusion surgery. Demographics, comorbidities, surgical factors, and postoperative outcomes were compared by IBS status. Logistic regression assessed the association between IBS and undergoing fusion after controlling for comorbidity and sociodemographic factors. Propensity score matching of pre- and intraoperative variables compared perioperative complications by IBS diagnosis.

**Results:**

Among 68,169 fusion patients, 2.1% had IBS. IBS patients were predominantly female (81.0% vs 53.7%, p < 0.0001) and similar in age to those without IBS. IBS patients had higher rates of nutritional deficiencies (5.4% vs 2.9%, p < 0.0001), electrolyte disorders (14.3% vs 11.5%, p = 0.001), arthritis (7.2% vs 4.3%, p < 0.0001), and osteoporosis (11.3% vs 5.8%, p < 0.0001) during admission. IBS was associated with increased odds (1.91, 95% CI[1.80, 2.02], p < 0.0001) of undergoing thoracolumbar fusion. After matching, IBS remained associated with increased odds of gastrointestinal complications (primarily ileus) (1.60, 95% CI[1.11, 2.33], p = 0.01) and prolonged stay (>3 days) (1.23, 95% CI[1.06, 1.43], p = 0.006).

**Discussion and conclusions:**

IBS was associated with increased surgical utilization, greater comorbidity, and modestly worse inpatient postoperative outcomes. These patients demonstrated higher rates of nutritional deficiency and gastrointestinal complications, potentially due to dietary restrictions and inherent gastrointestinal irritability. IBS may represent a risk factor in thoracolumbar fusion and should be considered during preoperative counseling.

## Highlights

• IBS was associated with higher odds of thoracolumbar fusion utilization.
• Nutritional deficiencies were more common with IBS patients.
• IBS increased odds of postoperative GI complications, mainly ileus.
• IBS was associated with prolonged length of stay.

## Graphical abstract

**Figure:**
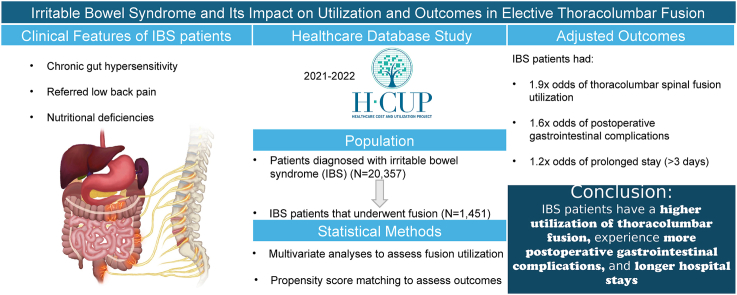

## 1 Introduction

Functional somatic syndromes (FSS), first described by Barsky et al. (Barsky and Borus, 1999), are a group of conditions characterized by physical symptoms and patient suffering without fully established pathophysiologic causes. In primary care, the prevalence of FSS is estimated to be as high as 34.8% (Haller et al., 2015). Some examples include irritable bowel syndrome (IBS),^1^ fibromyalgia (FM), chronic migraines, interstitial cystitis, and chronic fatigue syndrome. These conditions are commonly comorbid with psychiatric disorders, and patients with FSS often experience hyperalgesia more frequently than those without FSS (Barsky and Borus, 1999; Masood et al., 2024).

These features are particularly concerning for patient populations requiring potent opioids, including those undergoing spinal fusion. Recent studies have found that FSS – namely FM and chronic migraines – lead to worse outcomes in spine patients. Apart from greater postoperative opioid use, these patients more frequently experience postoperative complications and cite relatively inferior improvement in patient-reported outcome measures (PROMs) (Masood et al., 2024; Ablin et al., 2017; Donnally et al., 2019; Weiner et al., 2021).

Increasing attention has been placed on functional gastrointestinal disorders, which include IBS. IBS is a chronic and disabling syndrome that affects more than 10 to 15% of people in the US and up to 10% worldwide (Canavan et al., 2014; Hungin et al., 2005). IBS is often linked with other psychological comorbidities like anxiety and depression, and these patients have been shown to respond to medical/surgical neuromodulation in severe cases (Fadgyas Stanculete et al., 2021; Jayasinghe et al., 2023; Staudacher et al., 2023; Rana and Knezevic, 2013; Bieze et al., 2024). Interestingly, patients with IBS have been shown to undergo spine surgery 50% more often than those without IBS, potentially due to increased surgical referrals from nonspecific back pain (Longstreth, 2007; Longstreth and Yao, 2004). Nevertheless, there is minimal spinal literature describing the association of IBS with both patient presentation and postoperative outcomes. The aims of this study are thus threefold: 1) to identify the prevalence of IBS patients undergoing spinal fusion, 2) characterize common spinal pathology and surgical features among patients with IBS, and 3) compare inpatient complications by presence of IBS.

## 2 Methods

### 2.1 Data source

The Healthcare Cost and Utilization Project (HCUP) National Inpatient Sample (NIS) is the largest publicly available inpatient database in the United States. Between 2021 and 2022, the NIS tracked 13 million unweighted and 95 million weighted discharges from 48 states and 4554 hospitals. Each discharge included key demographic data, with primary and secondary diagnoses/procedures assigned using ICD-10 (International Classification of Diseases, Tenth Revision) codes.

### 2.2 Patient selection

The NIS was retrospectively reviewed between 2021 and 2022 to identify adult (≥18 years old) patients who underwent elective, primary thoracolumbar spinal fusion using ICD-10 procedural codes listed in Table 1. Patients who had active infection or underwent spinal fusion for tumor or trauma during the index admission were excluded. Patients with any form of IBS (constipation predominant (IBS-C), diarrhea predominant (IBS-D), mixed constipation and diarrhea subtype (IBS-M), or unspecified (IBS-U)) were extracted from the spinal fusion cohort using the dedicated ICD-10 codes as mentioned in Table 1.

**Table 1:** Study international classification of diagnosis edition 10 codes.

Study ICD10 Codes	
**Operative Approach**	
Thoracolumbar, Single Level - Anterior	0RGA070, 0RGA0A0
Thoracolumbar, Single Level - Posterior	0RGA071, 0RGA07J, 0RGA0A1, 0RGA0AJ
Thoracic, 2–7 Levels - Anterior	0RG7070, 0RG70A0
Thoracic, 2–7 Levels - Posterior	0RG7071, 0RG707J, 0RG70A1, 0RG70AJ
Thoracic, 8+ Levels - Anterior	0RG8070, 0RG80A0
Thoracic, 8+ Levels - Posterior	0RG8071, 0RG807J, 0RG80A1, 0RG80AJ
Lumbar, Single Level - Anterior	0SG0070, 0SG00A0
Lumbar, Single Level - Posterior	0SG0071, 0SG007J, 0SG00A1, 0SG00AJ
Lumbar, 2+ Levels - Anterior	0SG1070, 0SG10A0
Lumbar, 2+ Levels - Posterior	0SG1071, 0SG107J, 0SG10A1, 0SG10AJ
**Perioperative Complications**	
**Cardiac**	I43, I50, I09.9, I11.0, I13.0, I13.2, I25.5, I42.0, I42.5, I42.6, I42.7, I42.8, P29.0
**Respiratory**	I26.02, I26.09, I26.92, I26.93, I26.94, I26.99,J81.0,J80,J12.0, J12.1, J12.3, J12.4, J12.81, J12.89, J12.9,J13, J14, J15.0, J15.1, J15.20, J15.211, J15.212, J15.29, J15.3, J15.4, J15.5, J15.6, J15.7, J15.8, J15.9, J16.0, J16.8, J18.0, J18.1, J18.2, J18.8, J18.9,J69,J95.851,J96.00, J96.01, J96.02, J96.20, J96.21, J96.22, J96.90, J96.91, J96.92,T79.1,J95.2, J95.3,J95.811,J95.821, J95.822, T81.82, J95.84, J95.88, J95.89
**Gastrointestinal**	Ileus (K56.0, K56.7), Other postprocedural gastrointestinal complications (K91.89), Dysphagia (R13.1)
**Urinary**	N17.0, N17.1, N17.2, N17.8, N17.9, N30.0, N30.8, N30.9, N39.0, N99.0, N99.89
**Neurologic (ischemic or hemorrhagic stroke)**	I61.,I63., I97.811, I97.821
**Systemic Infection**	A41.01, A41.02, A41.1, A41.2, A41.3, A41.4, A41.50, A41.51, A41.52, A41.53, A41.59, A41.81, A41.89, A41.9, R65.20, R65.21, T81.44, T81.12
**Deep venous thrombosis**	I82.4
**Neurological Injury**	G83.82, G83.81, S15.15, G83.2, G83.3, G83.0, G82.5, G82.2, G82.21, G82.22, G82.20 G83.89, G83.9, G83.83, G83.10, S34.3
**Dural Tear**	G97.41, G96.11
**Mechanical Injury**	T84.216, T84.226, T84.296, T84.418, T84.428, T84.498, M61.00, M61.08, M61.09, M61.20, M61.28, M61.29, M61.40, M61.48, M61.49, M61.50, M61.58, M61.59, M61.9
**Fusion** **Disorders**	M96.0, M50.2, M50.3, M50.8, M50.9
**Acute Anemia**	D62
**Wound Infections**	T81.40, T81.41, T81.42, T81.43, T81.49, T81.60, T84.63, T84.7, M46.30, M46.31, M46.32, M46.33, M46.39, M46.40, M46.41, M46.42, M46.43, M46.49, M46.50, M46.51, M46.52, M46.53, M46.59, M46.20, M46.21, M46.22, M46.23, G06.1
**Other wound Complications**	T81.30, T81.31, T81.32, G97.63, L89.90, L89.139, L89.149, L89.159, L89.819, L89.899, L89.15, L89.81, L89.810, L89.95, L89.150 G97.51
**Irritable Bowel Syndrome Codes**	
K58.0, K58.1, K58.2, K58.8, K58.9	

### 2.3 Preoperative variables

Preoperative variables included age, sex, insurance status, and median household income. Comorbidity burden at the time of index admission was determined based on the presence of ICD-10 codes associated with those reported by Elixhauser et al. (1998) and Diagnosis Related Groups (DRG). Demographic and clinical characteristics were recorded.

### 2.4 Inpatient surgical characteristics

Surgical characteristics based on ICD-10 procedural codes, including approach and number of vertebrae fused, were recorded.

### 2.5 Outcome variables

The primary outcomes were inpatient complications listed in Table 1 (Im et al., 2024). Secondary outcomes, including length of stay and discharge disposition, were also listed. Prolonged length of stay was defined as length of stay greater than the population median of 3 days. In accordance with HCUP guidelines, cell counts less than or equal to ten were suppressed. Additional cells were suppressed when necessary to prevent back-calculation of suppressed values.

### 2.6 Statistical analysis

Chi-squared, logistic regression, and independent t-tests were used to identify differences in patient presentation, inpatient surgical characteristics, and postoperative outcomes between patients with and without IBS. Furthermore, 1:1 propensity score matching controlling for all preoperative and intraoperative variables was utilized to compare the impact of IBS on inpatient complication rates, length of stay, and discharge disposition. Each matched cohort had a sample size of 1420 patients. Statistical analyses were all conducted using SAS (Statistical Analysis Software) 9.4.

## 3 Results

### 3.1 Prevalence of and correlation of IBS among patients who receive spine surgery

Of 13,245,189 unweighted inpatient hospitalizations between 2021 and 2022, there were 68,169 thoracolumbar spinal fusion patients. Within this population, 1451/68,169 (2.1%) had IBS (Table 3). After adjusting for age, socioeconomic characteristics and disease-related risk of mortality, an IBS diagnosis was associated with increased odds (1.91, 95% CI[1.80, 2.02]) of elective thoracolumbar fusion (Table 2) compared to patients without IBS.

**Table 2:** Multivariate logistic regression analyses identifying predictors of elective thoracolumbar fusion.

Predictors of Elective Thoracolumbar Fusion		
	Odds Ratio [95%CI]	P-Value
IBS^1^ Diagnosis	1.91 [1.80, 2.02]	P < 0.0001
Age	1.04 [1.04, 1.04]	P < 0.0001
Female	0.79 [0.77, 0.80]	P < 0.0001
**Insurance Status**		
Medicare	Reference	P < 0.0001
Medicaid	0.62 [0.59, 0.64]	P < 0.0001
Private Insurance	1.16 [1.13, 1.19]	P < 0.0001
Self-Pay	0.43 [0.39, 0.47]	P < 0.0001
Other	1.73 [1.67, 1.79]	P < 0.0001
**Race**		
White	Reference	
Black	0.86 [0.84, 0.89]	P < 0.0001
Hispanic	0.67 [0.65, 0.69]	P < 0.0001
Asian or Pacific Islan.	0.51 [0.48, 0.55]	P < 0.0001
Native American	1.06 [0.95, 1.18]	P = 0.3
Other	0.74 [0.70, 0.78]	P < 0.0001
**Median Household Income**		
1st Quartile	Reference	
2nd Quartile	1.11 [1.09, 1.14]	P < 0.0001
3rd Quartile	1.16 [1.13, 1.19]	P < 0.0001
4th Quartile	1.17 [1.14, 1.20]	P < 0.0001
**Patient Location**		
Urban	Reference	
Large Metro	1.05 [1.03, 1.07]	P < 0.0001
Medium Metro	1.02 [1.00, 1.04]	P = 0.2
Small Metro	1.00 [0.96, 1.03]	P = 0.7
Micropolitan	0.97 [0.94, 1.00]	P = 0.08
Rural	0.82 [0.79, 0.84]	P < 0.0001
**All Patient Refined DRG: Risk of Mortality**		
Minor Likelihood of Dying	Reference	
Moderate Likelihood of Dying	0.39 [0.38, 0.40]	P < 0.0001
Major Likelihood of Dying	0.22 [0.21, 0.23]	P < 0.0001
Extreme Likelihood of Dying	0.19 [0.18, 0.20]	P < 0.0001

**Table 3:** Demographics and clinical characteristics comparing IBS cohort to non-IBS cohort.

Demographics and Clinical Characteristics			
**Variables**	**IBS^1^ Cohort, N (%); Total N = 1451**	**Non-IBS Cohort, N (%); Total N = 66,718**	**P-Value**
Mean Age	64.1 ± 11.0	64.0 ± 11.9	P = 0.5
Female	1175 (81.0%)	35,793 (53.7%)	P < 0.0001
Race	1258 (88.3%)	52,975 (81.3%)	P < 0.0001
White	83 (5.8%)	5360 (8.2%)	Missing = 1622 (2.4%)
Black	45 (3.2%)	3947 (6.1%)	
Hispanic	12 (0.8%)	1011 (1.6%)	
Asian or Pacific Islander	supressed	366 (0.6%)	
Native American	supressed	1463 (2.3%)	
Other	Missing = 26 (1.8%)	Missing = 1596 (2.4%)	
Insurance Status		36,015 (54.0%) 3947 (5.9%) 22,132 (33.2%) 404 (0.6%) 4124 (6.2%) Missing = 81 (0.1%)	P = 0.004 Missing = 85 (0.1%)
Medicare	819 (56.6%)		
Medicaid	73 (5.0%)		
Private Insurance	493 (34.1%) supressed		
Self-Pay	supressed		
Other	Missing = 4 (0.3%)		
Median Household Income	276 (19.3%) 354 (24.7%) 409 (28.6%) 393 (27.4%) Missing = 19 (1.3%)	14,339 (21.7%) 16,863 (25.6%) 17,386 (26.4%) 17,402 (26.4%) Missing = 728 (1.1%)	P = 0.06 Missing = 747 (1.1%)
(Lowest) 1st Quartile 2nd Quartile 3rd Quartile (Highest) 4th Quartile			
Fluid and Electrolyte Disorders	207 (14.3%)	7658 (11.5%)	P = 0.001
Nutritional Deficiency	79 (5.4%)	1947 (2.9%)	P < 0.0001
Coagulopathy	55 (3.8%)	2471 (3.7%)	P = 0.9
Rheumatoid Arthritis	105 (7.2%)	2879 (4.3%)	P < 0.0001
Hypothyroidism	421 (29.0%)	11,541 (17.3%)	P < 0.0001
Obesity	421 (29.0%)	18,256 (27.4%)	P = 0.2
Osteoporosis	164 (11.3%)	3902 (5.9%)	P < 0.0001
Uncomplicated Diabetes	159 (11.0%)	8970 (13.4%)	P = 0.006
Depression	475 (32.7%)	12,668 (19.0%)	P < 0.0001
Alcohol Abuse	12 (0.8%)	745 (1.1%)	P = 0.3
All Patient Refined DRG^a^: Risk of Mortality			P < 0.0001
Minor Loss of Function	1085 (74.8%)	53,250 (79.8%)	
Moderate Loss of Function	287 (19.8%)	9703 (14.5%)	
Major Loss of Function	62 (4.3%)	2787 (4.2%)	
Extreme Loss of Function	17 (1.2%)	976 (1.5%)	

a Disease Related Groups.

### 3.2 Demographics Comparisons

Patients with IBS were primarily female (1175/1451 [81.0%] vs 35,793/66,702 [53.7%], p < 0.0001), however there was no significant age difference (64.1 ± 11.0 vs 64.0 ± 11.9, p = 0.5). A slightly greater proportion of IBS patients had Medicare/Medicaid (892/1447 [61.6%] vs. 39,962/66,637 [60.0%], p = 0.004) and were of white race (1258/1425 [88.3%] vs. 52,975/65,122 [81.3%], p < 0.0001). There was no significant difference in median household income quartile between groups (Table 3).

### 3.3 Preoperative comorbidity

Patients with IBS presented more often with fluid and electrolyte disorders (207/1451 [14.3%] vs 7658/66,718 [11.5%], p = 0.001), more nutritional deficiencies (79/1451 [5.4%] vs 1947/66,718 [2.9%], p < 0.0001), more arthritis (105/1451 [7.2%] vs 2879/66,718 [4.3%], p < 0.0001), more hypothyroidism (421/1451 [29.0%] vs 11,541/66,718 [17.3%], p < 0.0001), more osteoporosis (164/1451 [11.3%] vs 3902/66,718 [5.8%], p < 0.0001), and less uncomplicated diabetes (159/1451 [11.0%] vs 8970/66,718 [13.4%], p = 0.006) compared to patients without IBS. A greater proportion of patients with IBS suffered from moderate to extreme risk of mortality due to overall comorbidity status (366/1451 [25.2%] vs 13,466/66,718 [20.2%], p < 0.0001) (Table 3). IBS patients had more depression (475/1451 [32.7%] vs 12,668/66,718 [19.0%], p < 0.0001) compared to patients without IBS. There was no significant difference in alcohol abuse, or coagulopathy between groups.

### 3.4 Comparison of primary spinal pathology

Patients with and without IBS suffered from similar primary spinal pathology necessitating thoracolumbar fusion. The most common spinal pathology listed was lumbar spinal stenosis followed by spondylolisthesis, lumbar radiculopathy, and lumbar disc degeneration (Table 4).

**Table 4:** Comparison of top 4 primary diagnosis by ICD10 codes based on presence of IBS Diagnosis.

Comparison of Primary Spinal Pathology			
	**IBS^1^ Cohort, N (%); Total N = 1451**	**Non-IBS Cohort, N (%); Total N = 66,718**	**P-Value**
**Lumbar/Lumbosacral Spine Pathology**	678 (46.7%) 220 (15.2%) 176 (12.1%) 21 (1.4%) Missing = 0 (%)	31,384 (47.0%) 10,057 (15.1%) 8769 (13.1%) 1172 (1.8%) Missing = 1 (0.001%)	P = 0.3 Missing = 1 (0.001%)
M48061, M48062 -Spinal Stenosis M4316 -Spondylolisthesis M5116, M4726 -Lumbar Radiculopathy M5136 - Disc Degeneration			

### 3.5 Perioperative characteristics

There was no significant difference in surgical approach or levels fused based on an IBS diagnosis (Table 5). An IBS diagnosis was significantly associated with prolonged length of stay (>3 days) before (646/1451 [44.5%] vs 25,386/66,718 [38.0%], p < 0.0001) and after 1:1 propensity score matching (636/1420 [44.8%] vs 563/1420 [39.6%], p = 0.006) (Table 6). Originally, fewer patients with IBS had routine home discharges (779/1451 [53.7%] vs 39,564/66,718 [59.3%], p = 0.0004). However, after 1:1 matching, IBS was no longer associated with discharge disposition (p = 0.4) (Table 6).

**Table 5:** Comparison of surgical approach based on presence of IBS Diagnosis.

Surgical Approach			
	**IBS^1^ Cohort, N (%); Total N = 1451**	**Non-IBS Cohort, N (%); Total N = 66,718**	**P-Value**
**Lumbar/Lumbosacral Spinal Fusion**			
Anterior Only Approach, Thoracolumbar Spinal Fusion	177 (12.2%)	7579 (11.4%)	P = 0.5
Combined Anterior Posterior Approach, Thoracolumbar Spinal Fusion	179 (12.3%)	7986 (12.0%)	
Posterior Only Approach, Thoracolumbar Spinal Fusion	1095 (75.5%)	51,153 (76.7%)	
**Intraoperative Levels Fused**			
Single Level	1036 (71.4%)	46,801 (70.1%)	P = 0.6
2-7 Levels	supressed	19,716 (29.6%)	
≥8 Levels	supressed	201 (0.3%)

**Table 6:** Comparison of inpatient complications based on IBS Diagnosis with propensity score matching results.

	IBS^1^ Cohort, N (%); Total N = 1451	Non-IBS Cohort, N (%); Total N = 66,718	P-Value; Odds Ratio	Propensity Matched IBS Cohort, N (%); Total N = 1420	Propensity Matched Non-IBS Cohort, N (%); Total N = 1420	P-Value
**Medical Complications**						
Neurologic (ischemic or hemorrhagic stroke)	supressed	133 (0.2%)	P = 0.6	supressed	supressed	P = 0.3
Cardiac	232 (16.0%)	10,849 (16.3%)	P = 0.8	223 (15.7%)	239 (16.8%)	P = 0.4
Respiratory	69 (4.8%)	2716 (4.1%)	P = 0.2	66 (4.6%)	74 (5.2%)	P = 0.5
**Gastrointestinal (ileus, dysphagia)**	76 (5.2%)	2298 (3.4%)	**P = 0.0002** **1.55 [1.22,1.96]**	74 (5.2%)	47 (3.3%)	**P = 0.01** **1.60 [1.11,2.33]**
Ileus Other postprocedural complications Dysphagia	62 (4.3%) 19 (1.3%) 12 (0.8%)	1830 (2.7%) 580 (0.9%) 433 (0.6%)		61 (4.3%) 18 (1.3%) 11 (0.8%)	40 (2.8%) supressed 14 (1.0%)	
Urinary	76 (5.2%)	3814 (5.7%)	P = 0.4	74 (5.2%)	82 (5.8%)	P = 0.5
Systemic Infection	23 (1.6%)	760 (1,1%)	P = 0.1	23 (1.6%)	14 (1.0%)	P = 0.1
Deep Venous Thrombosis	supressed	417 (0.6%)	P = 0.5	supressed	supressed	P = 0.8
Acute Posthemmorhagic Anemia	346 (23.9%)	13,621 (20.4%)	**P = 0.001** **1.22 [1.08,1.38]**	339 (23.9%)	327 (23.0%)	P = 0.6 1.05 [0.88,1.25]
**Surgical Complications**						
Neurological Injury	26 (1.8%)	1182 (1.8%)	P = 0.9	26 (1.8%)	22 (1.6%)	P = 0.6
Dural Tear	78 (5.4%)	3117 (4.7%)	P = 0.2	77 (5.4%)	64 (4.5%)	P = 0.2
Mechanical Injury	54 (3.7%)	2987 (4.5%)	P = 0.2	54 (3.8%)	72 (5.1%)	P = 0.1
Fusion Disorders	170 (11.7%)	7312 (11.0%)	P = 0.4	167 (11.8%)	141 (9.9%)	P = 0.1
Wound Infection	79 (5.4%)	3945 (5.9%)	P = 0.4	78 (5.5%)	91 (6.4%)	P = 0.3
Wound Complication	93 (6.4%)	3564 (5.3%)	P = 0.07	92 (6.5%)	82 (5.8%)	P = 0.4
**Length of Stay**			P**<0.0001**			**P = 0.006**
≤3 Days >3 Days	805 (55.5%) 646 (44.5%)	41,332 (62.0%) 25,386 (38.0%)	**Reference** **1.31 [1.18,1.45]**	784 (55.2%) 636 (44.8%)	857 (60.3%) 563 (39.6%)	**Reference** **1.23 [1.06,1.43]**
**Discharge Disposition**			**P = 0.0004**			P = 0.4
Home Home with Aide Non-Home	779 (53.7%) 422 (29.1%) 250 (17.2%)	39,564 (59.3%) 16,438 (24.6%) 10,551 (15.8%)	**Reference** **1.30 [1.16,1.47], P<0.0001** **1.20 [1.04,1.39], P = 0.01**	759 (53.4%) 417 (29.4%) 244 (17.2%)	791 (55.7%) 375 (26.4%) 252 (17.7%)	**Reference** 1.16 [0.98,1.38], P = 0.09 1.01 [0.82,1.23], P = 0.09

### 3.6 Inpatient complications among patients with irritable bowel syndrome

There was no difference in deep vein thromboses, neurological injury, fusion disorders, dural tears, wound infections, wound complications, mechanical complications, cardiac complications, respiratory complications, urinary complications, and cerebrovascular complications between groups. IBS was significantly associated with acute posthemorrhagic anemia (346/1451 [23.9%] vs 13,621/66,718 [20.4%], p = 0.0014) and gastrointestinal complications (primarily ileus) (76/1451 [5.2%] vs 2298/66,718 [3.4%], p = 0.0002). When controlling for preoperative and intraoperative differences through 1:1 propensity score matching, IBS remained significantly associated with gastrointestinal complications (74/1420 [5.2%] vs 47/1420 [3.3%], p = 0.01) (Table 5). Postoperative ileus was the most common gastrointestinal complication (1892/2374 [79.7%]), followed by other postprocedural gastrointestinal complications (599/2374 [25.2%]) and dysphagia (445/2374 [18.7%]) (Table 5). These diagnoses were not mutually exclusive, and patients could have more than one complication.

## 4 Discussion

### 4.1 Is there a theoretical association between IBS and thoracolumbar surgery?

Our multicenter analysis of a large sample size found that IBS was rare among patients undergoing thoracolumbar fusion, with a prevalence of ∼2%. However, IBS patients had nearly twice the odds (1.91, 95%CI [1.80-2.02], p < 0.0001) of undergoing elective thoracolumbar fusion, even after controlling for age, sociodemographic factors, and comorbidity burden. Our analysis supports earlier sentiments of increased spine surgery utilization among IBS patients by Longstreth and Yao (2004) (1.22, 95%CI [1.05-1.44], p < 0.001) and other gastroenterologists (Longstreth, 2007; Whorwell, 2004; Talley, 2004). However, their analysis did not account for salient predictors of spine surgery utilization, including rural versus urban residence, annual income, insurance status, comorbidity burden, or the type and extent of spine surgery (Longstreth, 2007; Im et al., 2024; John et al., 2018; Mannion et al., 2014).

**Fig. 1:**
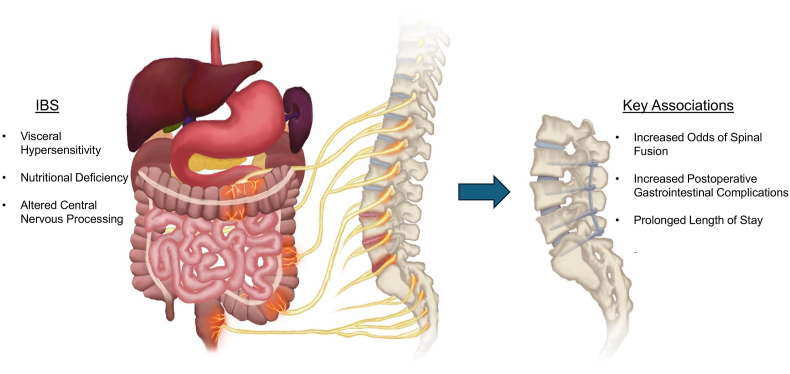
Figure depicting the association between irritable bowel syndrome (IBS) and perioperative complications.

Chronic low back pain is a common somatic manifestation of IBS, with prevalence ranging from 29% to 81% versus 3.9% to 20.3% in those without IBS (Shiha and Aziz, 2021; Azpiroz et al., 2000; Maxton et al., 1991). Increased peripheral and central sensitization to pain may be exacerbating these symptoms. When comparing 78 IBS patients and 57 healthy controls in the Veteran Affairs database, Zhou et al. (2010) found that IBS patients exhibited significantly lower response thresholds to cold, heat, ischemia, and pressure stimuli. Stabell et al. (2013) performed a similar study while controlling for psychological comorbidities (anxiety, depression) in a European cohort of 12,982 patients, and they also found reduced tolerance to noxious heat and cold stimuli among IBS patients. Moreover, the severity of IBS symptoms was indirectly associated with each patient's noxious tolerance threshold (Stabell et al., 2013). Referred back pain from vagal, splanchnic, and pelvic afferents innervating the lower GI tract, which converge with somatic afferents in the thoracolumbar dorsal root ganglia (DRG), may explain the rise in referrals for spine surgery amid increased pain sensitization (Fig. 1) (Stabell et al., 2013; Vermeulen et al., 2014).

### 4.2 Thoracolumbar pathology could influence IBS symptomatology

We found no correlation between the most common types of spinal pathology and IBS. However, degenerative spondylosis disrupting thoracolumbar DRG or lumbosacral splanchnic afferents may modulate colonic sensitivity and motility, which could lead to constipation, bloating, and abdominal pain that may be attributed to IBS. Mehrota et al. (Mehrotra et al., 2017), in a single institutional study with 64 patients, was the first to depict the resolution of preoperative constipation at 3 months after surgical decompression for cervicothoracic myelopathy, suggesting that constipation may serve as a modifiable symptom in degenerative spine disease.

Harrington et al. (2019) further showed anatomical connectivity between lumbosacral splanchnics and the spinal cord using retrograde fluorescent dye injection, with increased phosphorylated MAPK ERK 1/2 – a neuronal activation marker – observed in lumbosacral dorsal horn neurons following colonic distension in murine models. Naitou et al. (2015) expanded on these findings, demonstrating that intrathecal noradrenaline injection at L6-S1 enhanced colonic motility via modulation of lumbosacral efferents in rats.

In humans, sacral nerve modulation (SNM) has been explored as a treatment for visceral pain and colonic motility in chronic IBS (Bieze et al., 2024). Taken together, these findings suggest both anatomical and functional connections between the thoracolumbar spine and the lower gastrointestinal tract - connections that may be disrupted by degenerative spondylosis (Fig. 1).

### 4.3 IBS as a predictor of postoperative gastrointestinal morbidity

Our matched analyses showed that IBS was significantly associated with gastrointestinal complications, primarily postoperative ileus. We also found a small yet significant impact on prolonged length of stay. This novel finding suggests a potential impact of IBS on resource utilization and postoperative satisfaction. The causes of postoperative ileus after non-abdominal surgery are multifactorial and have been associated with increased age, prolonged operative time, drastic fluid and electrolyte shifts, and increased perioperative opioid usage (Lee et al., 2020; Artinyan et al., 2008). Emerging literature suggests that surgical stress triggers the hypothalamic release of corticotropin releasing factor (CRF). After binding to CRF receptors on gastrointestinal macrophages, this may activate a systemic inflammatory cascade (e.g.,toll-like receptors, cytokines) that contributes to gut inflammation and decreased motility (Hussain and Park, 2022). IBS patients may inherently have impaired neuroinflammatory responses that could be stimulated during the surgical manipulation and stress of thoracolumbar fusion. Supporting this concept, Bertilson et al. (2014) conducted a randomized study demonstrating how age-matched patients with IBS exhibited greater unilateral skin sensitivity to pain, heat, and cold in T7-L1 dermatomes, implicating neural involvement. The impaired neurogenic and stress-induced gastroinflammatory pathways may explain the increased chances of postoperative gastrointestinal complications. For IBS patients, interventions geared toward minimizing postoperative ileus including minimizing rapid fluid shifts, incorporating postoperative prokinetic agents, and early feeding may be warranted to limit further resource utilization and promote faster discharge (Sarmiento-Altamirano et al., 2025; Emile et al., 2024).

### 4.4 Preoperative nutritional optimization in IBS fusion patients

Though nutritional deficiencies among patients undergoing fusion were overall rare, IBS patients were almost twice as likely to have a nutritional deficiency during admission. In a meta-analysis of 24 studies, Bek et al. (2022) found that IBS patients often possessed lower levels of vitamin D, calcium, and iron compared to controls. These deficiencies were often attributable to exclusionary diets, like the low Fermentable Oligosaccharides, Disaccharides, Monosaccharides and Polyols (FODMAP) diet, which is recommended by the American College of Gastroenterology to minimize IBS symptoms (Bek et al., 2022; Lacy et al., 2021). Unfortunately, poor nutrition and frequent laxative use can worsen deficiencies in calcium, phosphate, and vitamin D, which are essential for protection against surgical infection, proper bone health, and spinal fusion.

A systematic review of multiple randomized controlled trials found that vitamin D supplementation significantly improved abdominal discomfort in IBS patients compared to placebo (Chong et al., 2022). In spine patients, Hu et al. (2022) found that vitamin D with concomitant calcium supplementation reduced time to spinal fusion and improved outcomes. Both studies suggest potential clinical benefits for IBS patients undergoing fusion surgery (Chong et al., 2022; Hu et al., 2022).

Together, these findings highlight the importance of preoperative dietary screening and appropriate laxative use among patients with IBS to optimize nutritional status, potentially minimizing postoperative complications associated with spinal fusion.

### 4.5 IBS fusion patients face higher overall resource utilization

Overall, our findings suggest a clinically meaningful increase in gastrointestinal complications (primarily postoperative ileus) and length of stay in IBS patients. Patients who develop postoperative ileus are more likely to report worse patient satisfaction, a finding compounded by their higher burden of psychiatric comorbidities (depression, anxiety) (Ebada et al., 2025). In addition, Merkow et al. (2020) found that postoperative ileus was associated with $10,205 in cumulative 30-day direct and indirect costs using multicenter hospital data from the U.S.

The potential for increased perioperative costs further exacerbates total financial burden among patients with IBS. Lacy et al. (2016), using a large retrospective claims database of 201,322 U.S. patients with IBS, reported that over one-third of patients with IBS required additional gastrointestinal specialist visits and underwent up to three unnecessary diagnostic tests or procedures – such as abdominal CT scans, ultrasounds, and colonoscopies – despite IBS being a clinical diagnosis.

Importantly, recent reviews by Chey et al. (2021) and Warner et al. (2025) highlight how integrated care for patients with IBS, including gastroenterology management, low-FODMAP therapy, and behavioral interventions, can significantly reduce symptom burden and healthcare utilization while improving patient satisfaction within three months compared to standard gastroenterologist care. For spine surgeons, recognizing IBS and preoperatively referring patients for interdisciplinary care may help promote enhanced recovery efforts after surgery, and ultimately reduce healthcare costs.

### 4.6 Limitations

This study has several limitations. First, our study relies on the presence of ICD-10 codes, which are subject to mischaracterization and may bias our results. Our selection strategy agrees with prior studies examining IBS in spine surgery populations, which have reported similar baseline characteristics (Longstreth and Yao, 2004; Bek et al., 2022). Second, the NIS lacks data on radiological findings, patient-reported outcomes, and readmissions data, limiting comprehensive contextualization of perioperative risk factors. Additionally, the retrospective cohort design precludes causal inference. However, the large, multicenter cohort of the NIS, which spans multiple calendar years, enhances the generalizability of our findings and underscores the clinical relevance of IBS as a preoperative comorbidity influencing the chances of undergoing spinal fusion and postoperative complications. Future studies incorporating PROMs, radiographic findings, and longitudinal gastrointestinal symptom assessments are necessary to further explore these relationships.

## 5 Conclusions

The presence of IBS in spine surgery patients is associated with a more complex clinical phenotype associated with worse outcomes. These patients face higher odds of spinal fusion compared to patients without IBS, a risk further compounded by nutritional deficiencies linked to dietary factors. Recognizing the symptomatic overlap of IBS is essential for spine surgeons, as extraintestinal referred pain may confound pain associated with spinal pathology, potentially leading to unnecessary surgery and increased postoperative gastrointestinal complications. For patients with IBS who require spinal fusion for structural causes of back pain, preoperative optimization - including identification and correction of nutritional deficiencies as well as interdisciplinary specialist referral to address both intestinal and extraintestinal symptoms - may reduce postoperative gastrointestinal complications and facilitate cost-effective, patient-centered care.

## Author contributions

Chiemela Izima and Andrew K. Chan contributed to study conception and design, data acquisition, statistical analysis, interpretation of data, and drafting of the manuscript. Farhan A. Khan, Bhargav Ayloo, Joshua S. Fuller, Nathan A. Shlobin, and Andrew K. Chan contributed to manuscript preparation. Isaac S. Lee contributed to figure and image creation and manuscript preparation. Dean Chou and Andrew K. Chan provided study supervision and contributed to manuscript review. All authors reviewed and approved the final manuscript.

## Ethical considerations

This study used de-identified data from the National Inpatient Sample (NIS) from the Healthcare Cost and Utilization Project (HCUP). Consequently, institutional review board approval and informed consent was not required.

## Data availability statement

The data used in this study are from the National Inpatient Sample (NIS), part of the Healthcare Cost and Utilization Project (HCUP), and are available from the Agency for Healthcare Research and Quality (AHRQ) upon reasonable request and completion of a data use agreement.

## Funding

This research did not receive any specific grant from funding agencies in the public, commercial, or not-for-profit sectors.

## Declaration of competing interest

The authors declare that they have no known competing financial interests or personal relationships that could have appeared to influence the work reported in this paper.

## Footnotes

1 Irritable Bowel Syndrome.
